# Students’ Inhibition, Cognitive Flexibility, and Performance Calibration Across Command, Practice, and Self-Check Teaching Styles in Physical Education: An Exploratory Field Study

**DOI:** 10.3390/jintelligence14080174

**Published:** 2026-08-01

**Authors:** Athanasios Kolovelonis, Ioannis Syrmpas

**Affiliations:** Department of Physical Education and Sport Science, University of Thessaly, Karies, 42100 Trikala, Greece; jsyrmpas@gmail.com

**Keywords:** cognition, metacognition, inhibition, cognitive flexibility, soccer

## Abstract

This exploratory study examined students’ acute responses in soccer performance, executive functions, and calibration accuracy to a single soccer session delivered using three different teaching styles (i.e., command, practice, and self-check) in physical education. One hundred and forty-seven students of four elementary schools attending four fifth-grade (80 students) and four sixth-grade (67 students) classes participated in the study. Two classes (one fifth- and one sixth-grade) from each elementary school were randomly assigned to one of the four groups of the study (i.e., self-check style, command style, practice style, and control group). Students in all experimental groups were taught the soccer-passing skill in a single 45 min session using identical activities and drills but different teaching styles. Control-group students attended a classroom-based session on the ancient Olympic Games. Students were pre- and post-tested in soccer-pass performance, estimation of soccer-pass performance, and design fluency test. Students in the self-check group demonstrated more favorable changes in inhibition and cognitive flexibility than students in the command- and practice-style groups, whereas students in the practice group also showed improvements in cognitive flexibility over time. No differences between groups were found in calibration accuracy. For soccer performance, although students in the self-check group improved from pre- to post-test, nonsignificant differences between groups were observed. The findings reflected students’ immediate responses under different instructional conditions during a single soccer session, rather than outcomes of the longitudinal learning process. These results are discussed in relation to the cognitive and metacognitive aspects of learning and performing sports skills in physical education, with particular emphasis on how students responded to different teaching styles.

## 1. Introduction

Physical education in school settings pursues multiple learning and performance goals in various domains (e.g., psychomotor, social, and cognitive) (A. Chen & Ennis, 2004). To achieve these goals, appropriate and effective teaching and learning methods should be adopted. This is because of the limited instructional time and the insufficient facilities or resources available in school physical education. In fact, using multiple styles of teaching may facilitate the achievement of a wide range of learning and performance outcomes (Garn & Byra, 2002).

### 1.1. The Spectrum of Teaching Styles

The Spectrum of Teaching Styles provides physical education teachers with a set of 11 teaching styles to help their students achieve a variety of diverse learning objectives (Byra, 2000; Mosston & Ashworth, 2008). Each style focuses on distinct objectives and outcomes and creates a different learning and instructional environment (Sanchez et al., 2012). From this perspective, the suitability of each teaching style depends on the specific learning or performance goals, the instructional context, and the characteristics of the students engaged in the learning process (Goldberger et al., 2012).

Spectrum teaching styles are usually organized in two clusters: the reproduction and production clusters (Kulinna & Cothran, 2003; Mosston & Ashworth, 2008). Within the reproduction cluster of teaching styles (i.e., command, practice, reciprocal, self-check, and inclusion), students engage in reproducing knowledge and practicing skills that have been prepared, demonstrated, and structured by the physical education teacher. Within the production cluster of teaching styles (i.e., the guided discovery, convergent discovery, divergent discovery, individual program, learner-initiated, and self-teaching styles), students engage in producing or discovering knowledge and skills, often working under problem-solving conditions that require exploration and independent decision making.

This study focused on three reproductive teaching styles. The focus was on the self-check style, while the command and practice styles were used as comparison teaching conditions, as these are the styles of teaching that physical education teachers use more often (Cothran et al., 2005). In the command style, students follow the teacher’s cues and instructions, trying to perform tasks, activities or skills presented by the teacher as accurately and quickly as possible. The teacher may stop the activity at any point to provide corrective feedback when needed, ensuring accurate and uniform performance. In the practice style, students can practice tasks independently after the teacher demonstrates them. The teacher decides the content and the structure of the lesson, but students are involved in making decisions, such as where to position themselves for practice, the order of the tasks, the intervals between activities, and when to start or stop each activity. During practice, the teacher observes students, answers their questions, and provides them with individual feedback when needed. The self-check style allows students to work and practice independently while performing tasks by following the instructions provided in a task sheet prepared by the teacher. Students monitor, record, and compare their performance against the criteria listed on the task sheet, giving themselves feedback to improve their learning and performance. During practice, the teacher observes and provides guidance only on whether students are using the self-check process correctly, if needed (Byra, 2000; Mosston & Ashworth, 2008).

Research in sport and physical education fields using various motor and sports skills has shown that teaching styles are associated with students’ learning and performance. For example, an early study found that the practice, the reciprocal, and the inclusion styles had positive effects on students’ skills in hockey (Goldberger et al., 1982). Elementary students who used the self-check and the reciprocal styles enhanced their basketball chestpass (Digelidis et al., 2018; Kolovelonis et al., 2011) and basketball dribbling and jump shot (Digelidis et al., 2018). The use of the self-check style helped fifth and sixth graders to improve their motor performance and increase their positive attitudes toward tennis compared to their counterparts who practiced with the command style (Patmanoglou et al., 2008). Moreover, the reciprocal and the self-check styles, rather than the command style, helped university students to effectively learn Greek folk dances (Pitsi et al., 2023). Furthermore, a systematic review and meta-analysis including six related studies showed that both the practice and the reciprocal styles of teaching had significant effects on students’ performance, with the practice style having the largest effects (Chatoupis & Vagenas, 2018).

Some evidence regarding how different teaching styles influence students’ motivation and metacognition has also been reported. Junior high students who were taught basic basketball skills (chest-pass, dribbling, and jump shot) for six weeks using the self-check style reported higher intrinsic motivation and identified regulation than those taught using the reciprocal style (Digelidis et al., 2018). Sixth-grade students who participated in sessions with the self-check style compared to those who were involved in the practice style reported higher scores in intrinsic motivation and metacognition (Papaioannou et al., 2012). Similarly, an intervention focusing on basketball, volleyball, and soccer sports skills delivered to seventh-grade students with a variety of student-centered teaching styles, such as the divergent discovery, the convergent discovery, the inclusion, the self-check, and the reciprocal styles, resulted in improved scores in intrinsic motivation, identified regulation, and metacognitive activities, compared to the comparison group (Chatzipanteli et al., 2015).

### 1.2. Executive Functions

Executive functions constitute a set of higher-order cognitive mechanisms and processes that can facilitate the regulation and the adaptation of goal-directed behavior, particularly in situations that are novel, demanding, or complex. Executive functions guide and supervise the processing of information necessary for purposeful and goal-directed actions that require a dynamic interplay of memory resources, attentional control, inhibitory processes, and self-regulatory capacities (Diamond & Ling, 2020). A consensus exists among experts that inhibition, working memory, and cognitive flexibility represent three core executive functions (Diamond, 2013). Inhibition denotes the capacity to deliberately suppress automatic, habitual, or unsuitable actions and responses in favor of more contextually appropriate solutions. It helps students to override strong internal predispositions in order to control their attention and regulate their behavior, thoughts, and emotional reactions in alignment with task demands or their long-term goals. Working memory involves the short-term holding and manipulation of information, helping students to integrate new pieces of information into their working plans, to follow complex instructions, and to translate them into action plans in real time. Cognitive flexibility denotes the ability to shift perspectives, allowing students to switch focus or attention between tasks or mental demands and to adapt strategies for facilitating problem-solving, adjusting in response to changing rules or environmental feedback. Cognitive flexibility is essential for problem-solving and for navigating dynamic learning contexts (Diamond, 2013, 2015; Diamond & Ling, 2020).

Executive functions serve as foundational processes supporting self-regulated learning and adaptive performance, especially in educational and achievement-oriented settings. Indeed, executive functions are critical for school success and academic achievement (Diamond & Ling, 2020; Nayfeld et al., 2013), success in sport (Vestberg et al., 2012), and self-regulation of learning and metacognitive control (Roebers, 2017). Various types of interventions have been tried to improve executive functions, including physical activity programs (Diamond & Ling, 2020; Pesce et al., 2021a; Pesce et al., 2021b). For example, physical activity interventions with cognitively enriched games and activities improved students’ executive functions (e.g., Kolovelonis & Goudas, 2023). However, not all physical activity programs have been found to be effective in enhancing executive functions (Diamond & Ling, 2020; Pesce, 2012). Interestingly, it has been hypothesized that the difference between successful and non-successful programs in improving executive functions may be partially based on the use of teaching methods that are effective in creating challenging learning environments that can promote students’ mental involvement and enhance their motivation to practice and to learn new skills (Pesce et al., 2016; Vazou et al., 2019). From this perspective, exploring the impact of diverse teaching styles on students’ executive functions is an area of significant interest.

### 1.3. Performance Calibration

A metacognitive variable that may be involved in students’ learning or performance is performance calibration, which represents the gap between actual and self-estimated performance. The closer this difference is to zero, the better calibrated the student is (Hattie, 2013). Calibration of learning and performance may affect students’ goals, motivation, metacognition, and self-regulation of learning (P. Chen & Rossi, 2013; Efklides & Misailidi, 2010). For instance, well-calibrated students may set more realistic goals, self-evaluate themselves more accurately, and generate accurate feedback, and thus they may regulate more effectively their learning or performance (Pieschl, 2009; Zimmerman & Moylan, 2009).

However, research evidence suggests that students are usually inaccurate when they are asked to estimate their performance in sports skills, tending to either overestimate or underestimate their actual performance. For example, a tendency to overestimate performance was found in golfers (Fogarty & Else, 2005), runners (Liverakos et al., 2018), and basketball players (McGraw et al., 2004). Moreover, studies in the field of physical education have shown that elementary students overestimated their performance in basketball dribbling, basketball chest pass, basketball shooting, and soccer pass (Kolovelonis & Goudas, 2012). However, an appropriately designed intervention, including, among others, goal setting and self-monitoring, may improve students’ calibration accuracy (Kolovelonis et al., 2022).

Performance calibration may have important implications for the effectiveness of teaching styles such as the self-check style, where students are involved in monitoring, recording, and evaluating their own performance and producing feedback for themselves. For example, moderate levels of accuracy were found among students practicing with the reciprocal or the self-check style. Most importantly, students who received more-accurate feedback (either peer feedback in the reciprocal style or self-feedback in the self-check style) outperformed their peers who received less-accurate feedback (Kolovelonis & Goudas, 2012).

### 1.4. The Present Study

Research on Spectrum teaching styles has mainly focused on the effects of the various teaching styles on students’ sport performance or skill acquisition (e.g., Kolovelonis et al., 2011; Patmanoglou et al., 2008) and motivational variables (e.g., Digelidis et al., 2018). Limited research has explored the associations of teaching styles with cognitive or metacognitive aspects of performance. For example, an intervention program had positive effects, among others, on students’ metacognitive activities (Chatzipanteli et al., 2015). However, this study included a variety of both productive and reproductive styles of teaching and, thus, distinct responses of each specific teaching style on students’ metacognition could not be isolated.

Therefore, the present study focused on cognitive (i.e., selected indicators of executive functioning, especially inhibition and cognitive flexibility) and metacognitive (i.e., calibration of performance) aspects of learning and performance when students are taught sports skills using different styles of teaching, in physical education. The main research question guiding the study was whether students would demonstrate different executive functions (i.e., inhibition and cognitive flexibility) and performance calibration responses under the self-check, command, and practice teaching styles. In particular, this study filled a gap in the Spectrum-related literature by exploring students’ acute cognitive-, metacognitive-, and motor-performance responses under these three teaching styles during a single physical education lesson. This evidence will increase our knowledge regarding the channels and the underlying cognitive and metacognitive mechanisms through which the Spectrum teaching styles may facilitate students’ learning and performance of sports skills during a single physical education session. Previous evidence has shown that teaching styles can acutely affect students’ sport performance in physical education (e.g., Kolovelonis et al., 2011), whereas acute effects on students’ executive functions have been observed following a physical education session with cognitively enriched games and activities (e.g., Kolovelonis & Goudas, 2023).

Among the three teaching styles examined in this study (command, practice, and self-check), the self-check style was expected to be associated with more favorable executive-function and performance-calibration responses. The self-check style, compared to the practice and the command styles, involves processes that are considered appropriate for the development of students’ executive functions and calibration of performance. In fact, during the self-check style, students work independently to master aspects of the skill, monitoring and checking their own performance against specific performance criteria, prepared by the teacher, which represent their learning or performance goals (Byra, 2000; Mosston & Ashworth, 2008). Previous empirical evidence has suggested that goal setting and self-recording of performance increased students’ executive functions more than simply practicing with feedback from the teacher (Samara et al., 2023). The practice style gives students control over elements of the practice such as practice pace, timing, and task intervals. This decision-making, along with the need to adjust or shift strategies, may influence their executive functions. In the command style, teachers make all instructional decisions while students simply follow instructions, reproducing skills and activities with minimal decision-making or cognitive engagement (Diamond, 2013; Diamond & Lee, 2011).

Setting specific learning and performance goals and monitoring and recording performance may also be associated with students’ performance calibration. These processes are inherent to the self-check style, but are absent in the practice or command styles. For example, self-monitoring of performance during practice with the self-check style may help students to trace and record information regarding their learning and performance and then to compare this evidence with the standards of performance, thus increasing their awareness of the current status of their performance (D. Ellis & Zimmerman, 2001). Furthermore, goal setting may increase students’ awareness of their performance and enhance the calibration accuracy of their performance (Hadwin & Webster, 2013).

This study explored students’ motor (soccer performance), cognitive (inhibition and cognitive flexibility), and metacognitive (performance calibration) responses under the self-check, command, and practice teaching styles in elementary physical education. It was hypothesized that differences would emerge among the teaching-style groups, with students in the self-check group demonstrating the most favorable responses in executive functions (i.e., inhibition and cognitive flexibility), performance calibration, and soccer-pass performance, followed by students in the practice group and then those in the command group. In addition, students in the experimental groups were expected to demonstrate more favorable outcomes than those in the control group.

## 2. Materials and Methods

### 2.1. Design

This study employed a group-randomized controlled trial to explore students’ motor (soccer-pass performance), cognitive (inhibition and cognitive flexibility), and metacognitive (calibration accuracy) responses under three different teaching styles (i.e., self-check, command, and practice) during a single soccer session in physical education. In particular, intact fifth- and sixth-grade classes were randomly allocated to one of the following groups: (a) Group 1 with the self-check style, (b) Group 2 with the command style, (c) Group 3, with the practice style, and (d) Group 4, a control group. Measures for soccer-pass performance, estimation of soccer-pass performance, and design fluency were involved before and after the soccer session.

### 2.2. Participants and Settings

Participants were 147 students (*M*age = 10.88, *SD* = 0.56, 72 boys, 75 girls) attending four fifth-grade (80 students) and four sixth-grade (67 students) classes of four elementary schools. In particular, Group 1 (self-check style) consisted of 38 students (19 boys), Group 2 (command style) of 34 students (16 boys), Group 3 (practice style) of 34 students (16 boys), and Group 4 (control group) of 41 students (21 boys). To enhance the ecological validity of the study, given that students are taught in intact classes at school, randomization was conducted first at the school level and then at the grade level. Specifically, schools were randomly allocated to one of the four groups of the study, and then two classes (one fifth-grade and one sixth-grade) from each elementary school were randomly selected. This procedure was adopted to prevent the leakage of experimental conditions between groups employing different teaching styles within the same school and to avoid having the same teachers teach groups assigned to different teaching styles. The elementary schools involved in this study were located in the same city and shared similar characteristics, including a comparable number of students and classes, similar sports facilities, and teachers with similar levels of teaching experience.

In Greece, fifth- and sixth-grade students attend two compulsory 45 min physical education lessons per week, taught by certified physical education specialists. The curriculum emphasizes the development of fundamental motor and sports skills and encourages the use of a variety of instructional approaches, including the Spectrum of Teaching Styles.

### 2.3. Measures

#### 2.3.1. Soccer-Pass Performance

Students’ soccer-pass performance was measured with a passing-accuracy test (Ali, 2011). In particular, students performed 10 passes, with no time limit, from a distance of 8 m, aiming to pass the ball through a target consisting of two cones 1 m apart and 0.5 m high. Students’ successful passes constituted their score in the test.

#### 2.3.2. Estimation of Soccer-Pass Performance

Just before performing the soccer-pass test, students estimated their soccer-pass performance by answering the following question: “How many passes out of 10 will you pass through the cones from this position?”

#### 2.3.3. Calibration Bias and Accuracy

The calibration accuracy index was computed for each student by subtracting actual soccer-pass performance from estimated performance and taking the absolute value. This index represents the magnitude of calibration error. Scores closer to zero represent greater calibration accuracy (Schraw, 2009). Calibration bias (i.e., the signed difference between estimated and actual performance) reflects the direction of calibration, but is not a linear indicator of accuracy. Therefore, it is not considered an appropriate measure for group mean comparisons (Griffin et al., 2013) or correlational analyses (Stankov et al., 2012). Therefore, calibration bias scores were used to classify students as accurate (score = zero), overestimators (positive scores), or underestimators (negative scores), indicating the direction of calibration.

#### 2.3.4. Executive Functions

The design fluency test, including three conditions measuring design fluency, inhibition, and cognitive flexibility, was used (Delis et al., 2001). The first condition of the test contains the core aspects of the design fluency skill, including working memory and creativity, whereas the second condition introduces an additional requirement for inhibiting automatic responses, and the third condition further incorporates demands on cognitive flexibility (Vestberg et al., 2020). In each condition, students had to draw, within 1 min, as many original designs as possible by linking dots with four sequential straight lines. The response sheet in each condition included 35 square boxes, each displaying an unstructured array of dots. In the first condition (i.e., design fluency), students were required to connect dots in boxes that contained five solid dots. In the second (i.e., inhibition) and third (i.e., cognitive flexibility) conditions, boxes contained five blank and five solid dots each, and students were required to connect only blank dots (condition 2) or to alternate between blank and solid dots, starting either from a blank or a solid dot (condition 3). Before completing each condition, students were instructed on how to perform the task, observed their physical education teacher demonstrate the test on the blackboard of the classroom, and then completed three boxes of dots as a trial. Students’ scores in each test condition reflected the number of correct, non-repetitive designs they produced. This test has been widely used to examine students’ executive functions in the fields of school physical education (e.g., Kolovelonis & Goudas, 2023) and sport (e.g., Vestberg et al., 2012, 2020).

### 2.4. Procedures

The University Ethics Review Committee and the local Directorate of Primary Education approved the design and the procedures of this study. The principals and the physical educators of each school provided their consent for conducting the study in their schools, while parental written consent was obtained for each student’s participation. Students participated in the study voluntarily, and their personal information was kept confidential. The physical education teachers from the three schools (i.e., three teachers, all male, with teaching experience ranging from 25 to 30 years) implemented the soccer sessions for all experimental groups during regular physical education hours in the schoolyards. In each school, the physical education teacher delivered the soccer session using a specific teaching style. Additionally, a fourth physical education teacher delivered a session on the ancient Olympic Games to the control-group students, which took place in their classroom. Before the implementation of the sessions, the physical education teachers were trained to deliver the lesson plan with the respective teaching style. The content and the teaching procedures for all experimental groups were described in detail in written lesson plans. These lesson plans were piloted in classes of students not involved in the main study. After the implementation of the session, the physical education teachers completed a record sheet including specific criteria reflecting the fidelity of the intervention. They recorded whether each activity in the session was delivered as planned and described in the written lesson plan, within the predefined time, and whether the principles of the respective teaching style were followed (e.g., whether students in the self-check style used their task sheets to record their performance). Based on these records, the session was delivered to each experimental group, as planned. One week before the field experiment, students were pre-tested on estimation of soccer-pass performance, soccer-pass performance, and the design fluency test. After the implementation of the soccer session, students were post-tested on the same variables, following the same procedures. Similar procedures were followed for the control-group students. During the week before the post-test, students of all groups had similar physical education experiences.

### 2.5. Description of the Experimental Conditions

The content of the soccer session was identical for all experimental groups. What was different was the teaching style used to deliver this content to the three experimental groups (see details below). The aim of the session was to help students learn to perform correctly the following three basic key points of the soccer-pass technique: (a) “put the support foot slightly bent next to the ball”, (b) “the toe of the support foot points in the direction of the pass”, and (c) “pass the ball with the inside of the foot”, and to increase their passing accuracy (Lennox et al., 2006).

The first part of the session included an 8 min organization and warm-up period. The second part of the session lasted 25 min and began with the physical education teacher providing students with oral instructions and a demonstration of the correct technique of the soccer pass (1 min). Next, students, in dyads, performed drills focusing on properly executing the technique of the pass (3 drills of 5 min each) and on increasing their accuracy in passing (3 drills of 3 min each). For example, in the first drill focusing on improving pass technique, students in dyads, at a 4–6 m distance, performed passes using a stationary ball placed on the ground. To increase their passing accuracy, students performed passes from a 4–6 m distance, aiming to pass the ball between two cones 1 m apart. The last part of the session included a modified small-scale soccer game (10 min) and a closing reflection and evaluation period (2 min) (R. Ellis, 2021; Lennox et al., 2006).

Students in the self-check style (Group 1) performed independently the drills described above, following instructions included in a task sheet provided by the physical education teacher. The sheet included the three basic key points of the technique of the soccer pass, a picture to illustrate these key points, an appropriate place for recording performance, and instructions on how to do it. By monitoring, recording, and comparing their performance against the performance standards described in the task sheet, students provided themselves with feedback to improve the technique and the accuracy of the soccer pass. During practice, the teacher provided students with feedback related to the appropriate use of the self-check style, if necessary. Students in the command style (Group 2) practiced the drills described above, following their physical education teacher’s instructions and cues, trying to perform the soccer pass correctly (in the first three drills) and accurately (in the last three drills). After the end of each drill, students received feedback from their teacher regarding their pass technique and accuracy. Students in the practice style (Group 3) performed the soccer drills presented by their teacher, selecting the location of their practice, the time devoted to each drill, and when to increase the distance from which they performed the pass. During practice, students received individual feedback from their teacher regarding their technique and accuracy in the soccer pass. Group-4 students (the control group) attended, in their classroom, a session regarding the ancient Olympic Games. This was a classroom-based, non-physical-activity control group, and it differed from the experimental groups in physical activity, instructional context, and arousal level. Therefore, it should be viewed as a reference condition, and comparisons with the experimental groups cannot be interpreted as reflecting teaching-style differences.

### 2.6. Statistical Analysis

Prior to analysis, the data were examined for accuracy of data entry, missing values, distributional fit, and univariate and multivariate outliers. To check for potential classroom nesting effects, unconditional linear mixed models were conducted for each post-test variable, to calculate Intraclass Correlation Coefficients (ICCs) [and Design Effects]. Because individual-level analyses were deemed appropriate, baseline differences between the four groups were examined with a one-way MANOVA for the design-fluency test conditions and with one-way ANOVAs for calibration accuracy and soccer performance. Differences among groups over time for the three conditions of the design fluency test were examined with a 4 (Group) × 2 (Time) repeated-measures MANOVA. Univariate tests, separate for each test condition, and comparisons between pre-test and post-test within each group, followed. Because baseline differences in calibration accuracy were observed among the four groups, group differences in post-test calibration accuracy were examined using a one-way ANCOVA, with Group as the factor, post-test calibration accuracy as the dependent variable, and pre-test calibration accuracy as the covariate. ANCOVA was selected to statistically adjust for baseline differences in calibration accuracy when examining group differences at post-test. Changes in soccer-pass performance across groups and time were tested with a 4 (Group) × 2 (Time) repeated-measures ANOVA, and comparisons between pre-test and post-test within each group were subsequently conducted. For all analyses and comparisons, the alpha level was set at 0.05. For estimating the size of the effects, the partial η^2^ and Cohen’s *d* were calculated (Cohen, 1988).

## 3. Results

### 3.1. Preliminary Analysis

Table 1 presents the means and standard deviations of students’ pre- and post-test scores for all dependent variables, separately, for the four groups of the study. Applying the criterion that kurtosis and skewness values divided by their standard errors should fall within ±1.96 to indicate normality (Field, 2024), almost all data for the dependent variables met the assumption of normal distribution. Some small deviations in skewness were found in the pre-test data regarding the estimation of soccer-pass performance in Group 1, in the first condition of the design fluency test (i.e., fluency) in Group 3, and in the post-test data for the second condition of the design fluency test (i.e., inhibition) in Group 4.

Preliminary cluster analysis using unconditional linear mixed models across the eight distinct classroom clusters (average cluster size = 18.38) revealed negligible nesting effects for the primary motor-performance and cognitive outcomes. Specifically, the intraclass correlation coefficients (ICCs) and corresponding design effects (*Deff*) were completely non-existent for soccer-pass performance (ICC = 0.000, *Deff* = 1.00) and inhibition (ICC = 0.000, *Deff* = 1.00), and exceptionally low for design fluency (ICC = 0.007, *Deff* = 1.12), and cognitive flexibility (ICC = 0.019, *Deff* = 1.33). In contrast, strong clustering occurred within the calibration accuracy measure, meaning classmates shared similar tendencies in performance calibration accuracy (ICC = 0.231, Deff = 5.01). Thus, the interpretation of the corresponding inferential tests for this variable should be approached with caution. A conditional random-intercept LMM was performed as a sensitivity analysis. However, the model failed to converge. The Hessian matrix was not positive definite, and the classroom intercept variance was zero, reflecting the sparse cluster structure. Despite this structural instability, the Group main effect remained statistically significant, *F* (3, 143) = 2.70, *p* = .048, with marginal *R*^2^ = 0.036, conditional *R*^2^ = 0.357, and an adjusted ICC of 0.333. This finding indicates that the descriptive trends across the instructional conditions reflect genuine raw data patterns, rather than artifacts of a single outlier classroom. The limited cluster structure (four schools, eight classes) is mathematically insufficient for stable multilevel estimation. Accordingly, these mixed-model indices should be interpreted with caution, supporting the adoption of individual-level analyses within an exploratory framework.

Baseline differences between the four study groups were examined for all dependent variables. The one-way MANOVA showed nonsignificant group differences in the three conditions of the design fluency test, *F* (9, 429) = 1.84, *p* = .06. Moreover, the one-way ANOVA showed significant differences between groups in the calibration accuracy, *F* (3, 143) = 4.02, *p* = .009, partial *η*^2^ = 0.078, and nonsignificant differences in the soccer-pass performance, *F* (3, 143) = 2.12, *p* = .10.

### 3.2. Group Differences in Inhibition and Cognitive Flexibility

Differences among the four groups in the three conditions of the design fluency test were examined with a 4 (Group) × 2 (Time) MANOVA. Multivariate outliers were assessed by calculating Mahalanobis distance values (Fidell & Tabachnick, 2003; Tabachnick & Fidell, 2019) and comparing them with the critical chi-square value at *p* < .001 with 6 degrees of freedom (corresponding to the six variables derived from the three design-fluency test conditions measured at pre-test and post-test), which was 22.46. No cases exceeded this criterion, indicating the absence of extreme multivariate outliers. Homogeneity of covariance matrices were tested with Box’s M test, *M* = 101.12, *F* (63, 45,880.78) = 1.48, *p* = .008. Considering that the sample sizes of four groups were unequal and the Box’s M test was nonsignificant at *p* < .001 (Tabachnick & Fidell, 2019), Pillai’s Trace was used as a more robust multivariate test (Field, 2024; Tabachnick & Fidell, 2019).

The 4 (Group) × 2 (Time) MANOVA with repeated measures showed a significant multivariate interaction for Group and Time (Pillai’s Trace = 0.174, *F* (9, 429) = 2.94, *p* = .002, partial *η*^2^ = 0.058) on students’ scores in the three conditions of the design fluency test (i.e., fluency, inhibition, and cognitive flexibility). Separately, for each test condition, repeated measures ANOVAs were followed. For condition 1 (i.e., fluency) a nonsignificant Group and Time interaction, *F* (3, 143) = 1.78, *p* = .15, but a main effect for Time, *F* (1, 143) = 34.19, *p* < .001, partial *η*^2^ = 0.19, were found. Significant Group and Time interactions were found for condition 2 (i.e., inhibition), *F* (3, 143) = 5.76, *p* < .001, partial *η*^2^ = 0.11, and condition 3 (i.e., cognitive flexibility), *F* (3, 143) = 4.37, *p* = .006, partial *η*^2^ = 0.08. Next, comparisons within groups were conducted, separately for the three conditions of the test, to check for differences between pre-test and post-test. In test condition 1 (i.e., fluency), significant pre-test-to-post-test improvements were found for Group 1 (self-check style), *t* (37) = 4.24, *p* < .001, *d* = 0.57, Group 2 (command style), *t* (33) = 3.17, *p* = .003, *d* = 0.37, and Group 3 (practice style), *t* (33) = 3.61, *p* < .001, *d* = 0.56, but not for Group 4 (control group), *t* (40) = 1.04, *p* = .30. In the second condition of the test (i.e., inhibition), significant pre-test-to-post-test improvements were found for Group 1 (self-check style), *t* (37) = 4.19, *p* < .001, *d* = 0.57, but not for Group 2 (command style), *t* (33) = 1.64, *p* = .11, Group 3 (practice style), *t* (33) = 1.66, *p* = .10, and Group 4 (control group), *t* (40) = −0.70, *p* = .49. In test-condition 3 (i.e., cognitive flexibility), significant improvements from pre-test to post-test were found for Group 1 (self-check style), *t* (37) = 3.54, *p* = .002, *d* = 0.40, and Group 3 (practice style), *t* (33) = 2.11, *p* = .043, *d* = 0.28, but not for Group 2 (command style), *t* (33) = 0.21, *p* = .83, and Group 4 (control group), *t* (40) = −1.34, *p* = .19.

### 3.3. Group Differences in Performance Calibration

Differences among the four groups in performance calibration were examined using a one-way ANCOVA to account for baseline difference between groups. The assumption of homogeneity of regression slopes was satisfied, as the interaction between the covariate (i.e., pre-test scores in calibration accuracy) and the independent variable (i.e., group) was nonsignificant *F* (3, 139) = 1.66, *p* = .178. After adjusting for the differences between groups in the calibration accuracy in pre-test, *F* (1, 142) = 5.39, *p* = .022, partial *η*^2^ = 0.037, the one-way ANCOVA, with Group as the factor, post-test calibration accuracy as the dependent variable, and pre-test calibration accuracy as the covariate, showed nonsignificant differences between groups in post-test calibration accuracy, *F* (3, 142) = 1.69, *p* = .172. The adjusted mean scores for calibration accuracy, along with the corresponding 95% confidence intervals (CIs), were 1.68 (95% CIs [1.16, 2.18]) for Group 1 (self-check), 2.21 (95% CIs [1.66, 2.76]) for Group 2 (command style), 2.10 (95% CIs [1.57, 2.64]) for Group 3 (practice style), and 1.48 (95% CIs [0.99, 1.97]) for Group 4 (control group).

Regarding the direction of calibration, the frequencies of accurate students, overestimators, and underestimators in all study groups at the pre-test and post-test are presented in Table 2. Overall, the frequency distributions suggested that, regardless of group, most students did not accurately estimate their soccer performance. Indeed, in the total sample, the percentage of students who accurately estimated their soccer performance was only 15% (22 students) on pre-test and 20% (29 students) on post-test. In contrast, 34% of the students (50 students, both at the pre-test and the post-test) estimated that their soccer performance would be lower than it actually was, whereas 75 students (51%) at pre-test and 68 students (46%) at the post-test estimated that their soccer performance would be higher than it actually was. Small variations in these frequencies from pre-test to post-test were also observed in almost all calibration-bias categories across the study groups. The frequency of accurate students remained stable in the self-check-style group, increased in the command-style and control groups, and decreased in the practice-style group. Overestimators decreased in the self-check style and control groups, remained unchanged in the practice-style group, and increased in the command-style group. Underestimators increased in the self-check style, practice-style, and control groups, but decreased in the command-style group. These data were not subjected to inferential statistical analyses because the primary analyses focused on the continuous measure of calibration accuracy, whereas the categorical classifications based on students’ bias scores were used solely for descriptive purposes. Given the relatively small number of cases in some categories, these patterns should be interpreted cautiously and considered descriptive in nature.

### 3.4. Group Differences in Soccer-Pass Performance

The 4 (Group) × 2 (Time) ANOVA with repeated measures and students’ score in the soccer pass performance as dependent variable showed a nonsignificant interaction for Group and Time, *F* (3, 143) = 1.72, *p* = .17, but a significant main effect for Time, *F* (1, 143) = 10.66, *p* < .001, partial *η*^2^ = 0.07. Within groups comparisons revealed significant improvement in soccer-pass performance from pre-test to post-test for Group 1 (self-check style), *t* (37) = 3.02, *p* = .005, *d* = 0.38. Nonsignificant improvements from pre-test to post-test were found in Group 2 (command style), *t* (33) = −0.33, *p* = .74, Group 3 (practice style), *t* (33) = 1.88, *p* = .07, and Group 4 (control group), *t* (40) = 1.82, *p* = .08.

## 4. Discussion

This study explored students’ cognitive (i.e., inhibition and cognitive flexibility), metacognitive (i.e., calibration accuracy), and motor (i.e., soccer performance) responses under different Spectrum teaching styles in physical education. Three groups of students were taught the soccer pass using a different teaching style (i.e., self-check, command, and practice style) during a single physical education session, whereas a control group of students who were taught a classroom-based session on the ancient Olympic Games were also involved. Students in the self-check group demonstrated greater improvements in inhibition and cognitive flexibility from pre-test to post-test than students in the command and practice groups. In addition, students in the practice group showed improvements in cognitive flexibility over time. The three experimental groups showed within-group improvements in design fluency. However, the interaction was nonsignificant, and thus this study cannot demonstrate significant differences among teaching styles regarding relative gains in design fluency. No differences between groups were found in calibration accuracy. Regarding soccer-pass performance, pre- to post-test improvements were found in the group of students who were taught the soccer pass with the self-check teaching style, without, however, outperforming the students of the other groups. Nonsignificant changes in all variables were found for the control group. Next, these findings are discussed in detail, drawing on prior empirical research and considering their theoretical and practical implications for the use of different teaching styles in physical education.

### 4.1. Inhibition and Cognitive Flexibility Under Different Teaching Styles

Students in the self-check group demonstrated greater improvements in inhibition and cognitive flexibility than students in the command and practice groups. Although the exploratory nature of the study precludes causal conclusions, this pattern of findings is consistent with our expectation that the self-check style may be associated with more favorable executive-function responses. The self-check style shifts responsibility for evaluation and decision-making from teachers to learners, enabling students to work independently to master skills, monitoring and checking their own performance against specific performance criteria (Byra, 2000; Mosston & Ashworth, 2008). Using the self-check style, students were involved in goal setting, monitoring, and reflection, adjusting their actions and strategies, if necessary, processes that are appropriate for strengthening cognitive flexibility and adaptive strategy shifting. During practice, students paused regularly to evaluate their performance, which likely inhibited automatic responses and suppressed impulsive execution in favor of deliberate monitoring, recording, and assessment, thereby activating inhibitory-control mechanisms (Diamond, 2013). Indeed, it has been found that goal setting and self-recording of performance increased students’ inhibition and cognitive flexibility more than simply practicing with feedback from the teacher (Samara et al., 2023), whereas reflective thinking and practice were associated with enhanced cognitive flexibility (Orakcı, 2021). However, all these proposed mechanisms should be directly examined and verified in future research.

Interestingly, students in the practice-style group demonstrated significant improvements in cognitive flexibility from pre-test to post-test. The practice style provides students with opportunities to be involved in making decisions regarding various aspects of the practice such as the pace and rhythm of practice, the start and stop times for each task, and the intervals between tasks (Byra, 2000, 2018). This partial decision-making responsibility requires students to monitor their practice, to avoid distractions, make adjustments, and shift strategies as needed, processes that are associated with students’ cognitive flexibility (Diamond, 2014; Diamond & Ling, 2020). In contrast, no significant changes in inhibition or cognitive flexibility were observed in the command-style group. Students in the command style had to follow their teacher’s cues and instructions, trying to reproduce the soccer-pass skills and activities as accurately as possible (Byra et al., 2014). The teacher made all instructional decisions, while students simply reproduced prescribed movements or responses with minimal decision-making, problem-solving, or cognitive engagement, thus limiting the potential to influence their executive functions (Diamond, 2013; Diamond & Lee, 2011).

Students in all experimental groups improved their scores on the first condition of the design fluency test. Since no differences emerged among the experimental groups, this improvement cannot be linked to a specific teaching style. Rather, the improvement may have resulted from participation in the soccer session itself. Indeed, control-group students who attended a classroom-based lesson on the history of the ancient Olympic Games did not demonstrate any improvement in the design fluency test. Nevertheless, the potential influence of task-familiarity and practice effects cannot be entirely excluded, given that the same measures were administered at pre-test and post-test. Although the absence of improvement in the control group suggests that repeated testing was unlikely to be the primary driver of the observed gains, the control condition differed from the experimental groups in terms of setting, arousal, and task context. The first condition of the test measures design fluency, requiring students to generate as many unique designs as possible. In doing so, they must use working memory to keep track of their previous responses and avoid repeating them. Thus, working memory and creativity are involved in the first condition of the design fluency test (Vestberg et al., 2020). Moreover, the capacity to generate novel designs has been found to rely primarily on motor planning, that is, the ability to produce novel motor actions (Suchy et al., 2010). Indeed, positive associations between physical activity and working memory (Russo et al., 2021) and creativity (Bollimbala et al., 2021) have been reported. However, these interpretations were not directly tested in the present study, and therefore they should be further explored and verified in future research.

This is the first study, to our knowledge, which explored students’ executive-function responses (i.e., inhibition and cognitive flexibility) under selected Spectrum teaching styles. Previous studies in physical education have focused on the content of the physical activity programs, showing that cognitively enriched physical-activity programs enhanced students’ executive functions (e.g., Kolovelonis & Goudas, 2023). This study extends previous research by suggesting that students in the self-check group, and, to a lesser extent, those in the practice group, demonstrated more favorable inhibition and cognitive flexibility responses than students in the other groups. These findings provide preliminary support for the hypothesis that some teaching methods may create challenging learning environments that are more conducive to students’ mental engagement during physical education lessons (Pesce et al., 2016; Vazou et al., 2019). Evidence from a similar line of research has also suggested that using the varied linear pedagogy approach that involved teaching with a reproductive teaching approach, reflected in the “self-check” and “reciprocal” styles, improved students’ inhibitory control (Invernizzi et al., 2022).

### 4.2. Performance Calibration Under Different Teaching Styles

Contrary to our hypothesis, no differences between groups were found in students’ calibration accuracy regarding soccer performance. Generally, students in this study, regardless of the group, did not accurately estimate their performance. The presence of this overestimation is consistent with previous empirical findings in school physical education suggesting that students usually overestimate their performance in various sports skills, including basketball dribbling, chest pass, and shooting, and soccer-pass (Kolovelonis & Goudas, 2012). Previous evidence also suggested that students who were taught with the reciprocal or the self-check style did not differ in calibration accuracy, but those who received more-accurate feedback during their practice with one of these styles outperformed those who received less-accurate feedback (Kolovelonis & Goudas, 2012). In this study, it was expected that involving students in goal setting, monitoring, and recording performance (e.g., during practice with the self-check style) would increase their awareness of learning and performance and enhance their calibration accuracy (D. Ellis & Zimmerman, 2001; Hadwin & Webster, 2013). However, this hypothesis was not confirmed. The single physical education session was not sufficient to meaningfully shift students’ calibration accuracy. Probably, improvements in calibration accuracy may need more time to occur and require multi-component interventions (Digiacomo & Chen, 2016; Gutierrez de Blume, 2022). Indeed, it has been found that an appropriately designed intervention, including not only goal setting and self-monitoring, but also self-talk, self-evaluation, self-reflection on performance, and causal attributions can help students increase the accuracy of their performance estimations (Kolovelonis et al., 2022). Moreover, baseline differences in calibration accuracy were observed, with students in the self-check style showing lower scores (indicating higher accuracy) compared to the other groups, which made further improvements more difficult.

### 4.3. Soccer Performance Under Different Teaching Styles

Students in the self-check-style group demonstrated a significant improvement in soccer-pass performance from pre-test to post-test. However, this within-group improvement did not translate into superior performance relative to the other groups, as nonsignificant between-group differences were observed. No significant pre-test-to-post-test improvements were found in the command- and practice-style groups. Previous findings have shown that the self-check style had positive effects on students’ sports performance. Indeed, students who practiced with the self-check style significantly improved their performance in a variety of sports skills, including basketball chest pass (Digelidis et al., 2018; Kolovelonis et al., 2011), basketball dribbling and jump shot (Digelidis et al., 2018), tennis skills (Patmanoglou et al., 2008), and folk dances (Pitsi et al., 2023). Moreover, the self-check style, compared to the command style, has been found to be more effective for university students to learn Greek folk dances (Pitsi et al., 2023). The self-check style involves students in goal setting and self-monitoring of their performance, processes that have been found to be effective in improving students’ motor and sports performance and in promoting self-regulated learning in physical education (Kolovelonis et al., 2022).

### 4.4. Practical Implications

From an applied perspective, the Spectrum of Teaching Styles offers physical education teachers a rich repertoire of teaching styles designed to help students achieve diverse learning objectives across multiple domains (Mosston & Ashworth, 2008). Each style focuses on distinct objectives and creates a unique instructional environment, enabling educators to shape learning experiences that best align with desired outcomes. Consequently, physical educators can select the most appropriate style by considering the specific characteristics of their students, the goals they wish to pursue, and the unique conditions of the teaching context, such as limited time, space, or available resources (Goldberger et al., 2012). Given that school physical education seeks to promote not only psychomotor, but also cognitive and social development, relying on a single instructional approach may be insufficient. Instead, employing the most appropriate teaching styles can more effectively address these multiple aims, facilitating a wide range of learning and performance outcomes despite the constraints often present in school settings (Garn & Byra, 2002).

The findings of this study suggest that the self-check style may be associated with more favorable responses in inhibition, cognitive flexibility, and soccer performance under the instructional conditions examined. This evidence may encourage physical educators to incorporate not only the command and practice styles (Chatoupis, 2018; Kulinna & Cothran, 2003), but also a broader range of teaching styles into their instructional repertoire. For example, by using the self-check style for teaching sports skills, physical educators can enhance both the psychomotor and cognitive aspects of their students’ learning and performance. The self-check style is also appropriate for involving students in self-regulated learning. Indeed, through the self-check style, students set learning and performance goals, monitor and evaluate their progress against performance standards, and improve their performance (Kolovelonis et al., 2022).

### 4.5. Limitations and Future Research

The study’s limitations should be acknowledged. For example, the intervention consisted of only a single session; therefore, the findings should be interpreted as reflecting students’ immediate responses under different instructional conditions, rather than long-term learning outcomes. These acute responses offer useful insight but do not necessarily indicate lasting cognitive, metacognitive, or motor changes. To determine whether these observed patterns persist or accumulate over time, future studies should use a longitudinal design involving multiple sessions and retention measures. In addition, this study involved only one sport skill (i.e., soccer pass), which was taught to students during a single physical education session. Future research may examine the effects of interventions with a larger number of sessions and a variety of sport and motor skills. Moreover, soccer performance was measured with a single test measuring the precision of students’ pass execution. Future research may focus on multiple indicators of sports performance, including technical aspects of motor- and sports-skills performance. Similarly, executive functions were measured with a single test (i.e., the design fluency test). The advantage of this test is that it can be easily and quickly administered at the class level. Future studies would benefit from employing multiple assessments to evaluate executive function. Another important limitation related to the study’s internal validity was that the fidelity of intervention implementation was based solely on teachers’ self-reported data recorded immediately after the intervention, and was not independently verified. Future research should also involve fidelity checks using external raters to ensure that the interventions are implemented as they are planned.

A further limitation is that students were nested within eight classes and clustering effects (assessed via ICCs) were negligible for most outcomes but more pronounced for calibration-related measures, suggesting that standard errors for these variables may be somewhat underestimated. This represents a major limitation in the interpretation of the calibration accuracy results. Moreover, with only eight classes, the available cluster structure was insufficient for reliable estimation, and more complex multilevel modeling was not feasible. Consequently, the inferential results should be considered exploratory and interpreted with caution. To assess the robustness of the findings, a post hoc conditional random-intercept LMM sensitivity analysis was performed. The analysis confirmed that the cluster structure (four schools and eight classes) was too sparse to support stable multilevel estimation, resulting in a non-positive definite Hessian-matrix warning and redundant classroom-level variance parameters. Except for the statistical instability introduced by sparse clusters, three contextual considerations justify the use of an individual-level analytic frame. First, preliminary checks showed that classroom-level nesting was negligible for the motor and cognitive outcomes central to our conclusions, becoming meaningful only for calibration accuracy, a variable for which no group differences emerged. Second, variation in teacher involvement across conditions is an inherent feature of Mosston and Ashworth’s (2008) framework, which defines teaching styles by who makes the decisions. Third, any potential teacher variation was minimized through standardized, pre-piloted lesson plans, which ensured identical content, activities, and timing across all groups. Furthermore, statistical analysis focused on the individual level rather than the group level, as the aim of the study was to explore students’ cognitive and behavioral responses under different instructional conditions. To enhance the ecological validity of the study, given that students are taught in intact classes at school, randomization was conducted first at the school level and then at the grade level. However, this design may have increased the risk that school-level factors influenced the findings, thereby representing a major threat to internal validity. Nevertheless, as a field-based study conducted under authentic physical education conditions, the design aimed to balance methodological control with ecological validity. The elementary schools involved in this study shared similar characteristics (e.g., number of students and classes, sport facilities, and teachers with similar teaching experiences), reducing, although not eliminating, the likelihood of substantial school-level differences. Furthermore, randomization at the school level prevented contamination between experimental conditions and avoided having the same teachers teach groups assigned to different teaching styles. In addition, all participating teachers had comparable teaching experience and received training in the implementation of the assigned teaching style prior to data collection.

Further research should examine the link between teaching styles and executive functions. This study focused on three teaching styles for teaching a soccer-passing skill, with all groups receiving identical session content except for the instructional approach. Considering, however, that the nature and the characteristics of the content of a physical activity program (cognitively enriched or not) play an important role in enhancing students’ executive functions (e.g., Kolovelonis & Goudas, 2023), the interplay between teaching styles and the type of content delivered through each style, with respect to their effects on students’ executive functions, warrants further investigation. Future research should also examine the effects of additional teaching styles on students’ executive functions, particularly through comparisons across the broader clusters of the Spectrum (direct styles, student-assessment styles, discovery styles, and student-initiated styles; Syrmpas et al., 2020). Motivational variables may also be involved in this research. These could include examining how different teaching styles influence students’ self-efficacy, enjoyment, or situational interest, given that these constructs play a central role in students’ engagement and learning processes. Such evidence would not only enrich our understanding of the motivational pathways activated by each teaching style, but also help clarify how these motivational responses interact with students’ cognitive, metacognitive, and psychomotor development.

## 5. Conclusions

This exploratory, field-based study provides preliminary evidence regarding students’ acute responses under the self-check, command, and practice teaching styles in physical education. Students in the self-check group demonstrated greater improvements in inhibition and cognitive flexibility from pre-test to post-test than students in the command and practice groups, while students in the practice group also showed improvements in cognitive flexibility over time. No differences among groups were observed in calibration accuracy. Moreover, students in the self-check group improved their soccer-passing performance from pre- to post-test, although their performance did not differ from that of the other groups at post-test. Taken together, these findings suggest that the self-check teaching style may be associated with more favorable acute cognitive and motor performance responses under the instructional conditions examined in the present study. However, given the exploratory nature of the study and the inability to fully separate teaching style, teacher, and school effects, the findings should be interpreted with caution. Further research using more rigorous experimental designs is needed to confirm and extend these findings.

## Figures and Tables

**Table 1:** Means and standard deviations for students’ pre- and post-test scores in all dependent variables, presented separately for each group of the study.

	Group 1 Self-Check Style				Group 2 Command Style				Group 3 Practice Style				Group 4 Control Group			
	Pre-Test		Post-Test		Pre-Test		Post-Test		Pre-Test		Post-Test		Pre-Test		Post-Test	
Variable	*M*	*SD*	*M*	*SD*	*M*	*SD*	*M*	*SD*	*M*	*SD*	*M*	*SD*	*M*	*SD*	*M*	*SD*
Soccer performance	4.39	2.33	5.24 *	2.19	5.03	2.52	4.94	2.57	3.82	2.07	4.56	2.12	3.93	2.03	4.46	2.34
Estimation of soccer performance	4.56	2.14	4.71	2.66	5.38	2.51	7.06	2.35	4.06	2.58	4.38	2.35	4.78	1.64	4.78	1.85
Calibration accuracy	1.47	1.54	1.58	1.35	2.59	1.14	2.35	2.07	2.00	1.69	2.12	1.45	1.73	1.29	1.44	1.48
Design fluency	6.16	3.20	8.00 *	3.29	5.85	3.64	7.26 *	3.90	7.53	3.24	9.41 *	3.47	7.76	4.20	8.29	4.39
Inhibition	6.18	3.68	8.13 *	3.11	7.03	3.66	7.68	3.02	7.41	2.86	8.03	2.66	8.02	3.98	7.83	3.59
Cognitive flexibility	4.18	3.18	5.47 *	3.29	4.88	3.06	4.97	3.69	3.59	3.06	4.38 *	2.59	4.76	3.46	4.37	3.32

* Significant within group difference compared to pre-post.

**Table 2:** Frequencies of accurate, overestimators, and underestimators for all groups of the study.

Group	Accurate		Overestimators		Underestimators	
	Pre-Test	Post-Test	Pre-Test	Post-Test	Pre-Test	Post-Test
Group 1: Self-check style	10	10	15	12	13	16
Group 2: Command style	0	3	20	27	14	4
Group 3: Practice style	7	4	14	14	13	16
Group 4: Control Group	5	12	26	15	10	14
Total	22	29	75	68	50	50

## Data Availability

The data underlying the results presented in the study are part of a research program and are available on request from the first author (AK; akolov@pe.uth.gr).
